# The Small Mammal Sequence from the c. 76 – 72 ka Still Bay Levels at Blombos Cave, South Africa – Taphonomic and Palaeoecological Implications for Human Behaviour

**DOI:** 10.1371/journal.pone.0159817

**Published:** 2016-08-10

**Authors:** Turid Hillestad Nel, Christopher Stuart Henshilwood

**Affiliations:** 1 Department of Archaeology, History, Culture and Religion, University of Bergen, Bergen, Norway; 2 Evolutionary Studies Institute, University of the Witwatersrand, Johannesburg, South Africa; Max-Planck-Institut fur Menschheitsgeschichte, GERMANY

## Abstract

The Still Bay, c. 76–72 ka, a prominent techno-tradition during the Middle Stone Age of southern Africa, has yielded innovative technologies, symbolic material culture, and shows evidence of expansion of hunting techniques and subsistence strategies. In this paper we present the results of the first systematic, taphonomic and palaeoenvironmental study of micromammals from the Still Bay levels at Blombos Cave. Our taphonomic analysis indicates that the micromammals were accumulated by avian predators occupying the cave. Post-depositional processes affecting the micromammal assemblage include organic waste decomposition and conditions associated with a limestone cave environment. The palaeoenvironmental reconstruction shows that Marine Isotope Stage (MIS) 5a at Blombos Cave had diverse micromammal communities occupying a variety of habitats and with rainfall pattern equal to present. The transition from MIS 5a to 4 is indicated by less diverse micromammal assemblages, increase in grassland and scrub vegetation, shifts in seasonal precipitation, and a decline in shrubs associated with fynbos. The onset of the glacial conditions associated with MIS 4 is visible in the micromammal assemblage. However humans occupying Blombos Cave during this c. 5 ka period showed an ability to cope with changing environmental conditions and were able to adapt and utilise a variety of available resources.

## Introduction

Climate, demography and resource procurement strategies are central factors presented as possible contributors to the visibility of behavioural modernity in the Middle Stone Age (MSA) of South Africa (e.g. [1–10]). The Still Bay (SB) industry c. 76–72 ka ([11]: also see Table 1), typified by bifacial foliate points [12], contains a rich record of material culture associated with early modern humans in the MSA [13–15].The SB sequence at Blombos Cave (BBC) have yielded artefacts such as engraved ochres, shell beads and polished bone tools that are regarded as indicators of symbolically mediated social behavior [11, 13–15]. Extensive faunal and other anthropogenic remains, such as intact hearths, show intensive utilization of the cave at this time [13–19].

**Table 1:** Dating of the overlying hiatus level and the M1 and M2 Upper phases comprising the Still Bay sequence at BBC.

*Phase*	*Layer*	*Age (ka)*	*Mean age phase (ka)*	*Method*	*Reference*
**Hiatus**	DUN	69 ± 5, 70 ± 5	68 ± 4	OSL	[42–45]
**M1**			73 ± 3	OSL	[42–43]
	CA	73.3 ± 4.4		OSL	[48]
		67 ± 7, 77 ± 8, 81 ± 10		TL	[46]
	CC	72.7 ± 3.1, 72.5 ± 4.6, 74.6 ± 3.9		OSL	[42–43, 48]
		68 ± 6, 82 ± 8		TL	[46]
	CD	74.9 ± 4.3		OSL	[48]
**M2 Upper**			77 ± 3	OSL	[44–45]
	CF	105 ± 9		TL	[46]
	CFA	69.7 ± 3.9		OSL	[48]
	CFB/CFC	68.8 ± 4.6, 75.5 ± 5.0		OSL	[48]
	CFD	76.8 ± 3.1, 76.7 ± 4.8		OSL	[44–45, 48]

The SB at BBC defines a highly innovative period, but similar to other MSA sites in the region, there appears to be a discontinuous record [9–10, 20–25] where crucial innovations emerge, diffuse and seemingly disappear. Several theories for the patchy appearance of innovative technologies and resource procurement strategies have been proposed (see [1, 6, 10–11, 26–29]). This emerging range of theories has highlighted the need for sources of information beyond material culture. Investigating the micromammals found in association with the human and non-human deposited layers at BBC hold promise of understanding the variable palaeoenvironments that *Homo sapiens* encountered during their SB occupation at this site. The suitability of micromammals as palaeoenvironmental informants is due to their small home ranges, precise ecological requirements and role as primary consumers in the food chain [30].

### General context and human occupation of Blombos Cave

**Fig 1:**
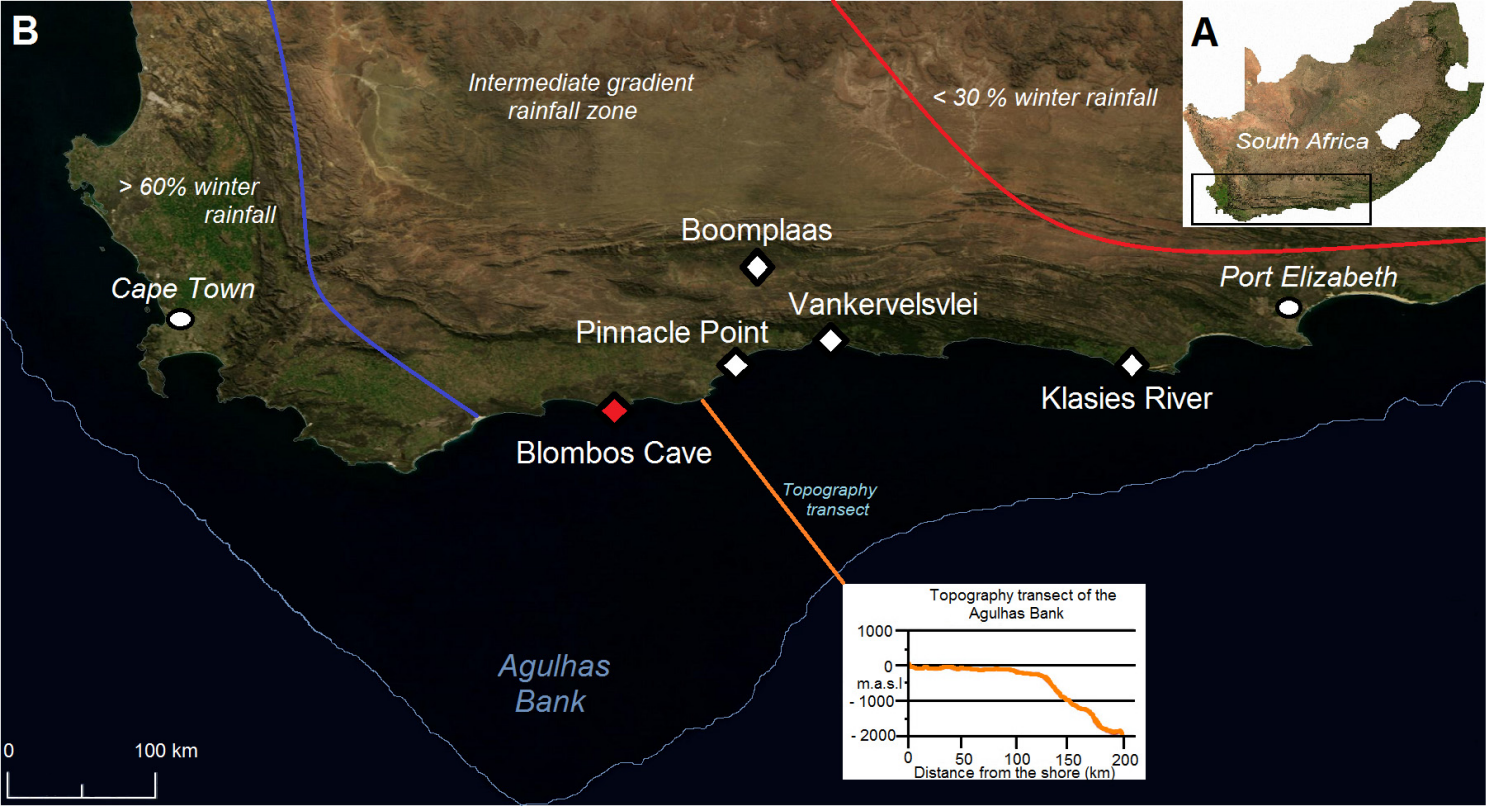
Location of BBC and other sites mentioned in the text. The blue and red lines indicate the approximate positions of the winter rainfall (WRZ) and summer rainfall zones (SRZ) respectively. The area between the contour lines is the intermediate gradient rainfall zone (YRZ), characterised by all year rainfall at transient intervals moving from west to east. The map includes a topography transect of the offshore platform marked in orange and based on data from Fischer et al. [32] (the topography transect of the offshore platform is similar but not identical to the original image from Bar-Matthews *et al*. [33] and is for illustrative purposes only). Satellite maps: A: Maplibrary.org (public domain): http://www.maplibrary.org/index.php. B: NASA Earth Observatory (public domain): http://earthobservatory.nasa.gov/.

**Fig 2:**
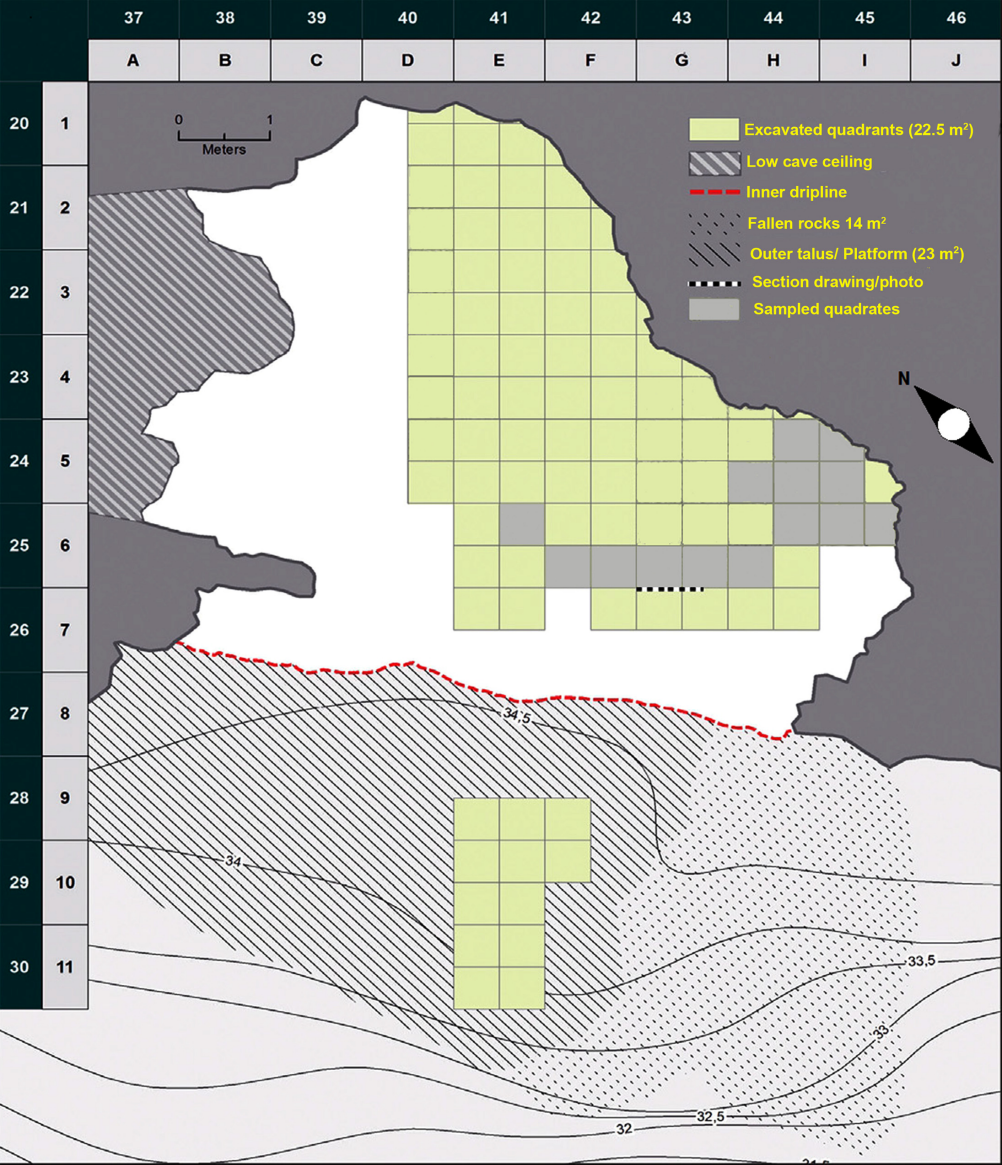
Site layout of BBC. The sampled quadrates for the study presented in this paper are marked in light grey. Modified with permission after [34].

BBC (34°24.54.58”S, 20°13’31.21”E) is a c. 55 m^2^ limestone cave situated in the Blombosfontein Nature Reserve (Hessequa Municipality) on the south coast of the Western Cape Province of South Africa approximately 300 km east of Cape Town (Figs 1 and 2). At present it is c. 100 m from the ocean and c. 35 m above sea level [17, 29]. The cave was formed as a result of solution action and wave cutting of the calcarenite and calcrete cliff that lies above a basal layer of Table Mountain Sandstone of the Cape Supergroup [17, 31].

### Current climate and vegetation

The interaction between the Southern Hemisphere tropical and temperate climate systems are the main cause of the current rainfall pattern in southern Africa [35]. BBC is situated in an intermediate gradient rainfall zone (YRZ), characterized by aseasonal rainfall with 54% precipitation during the winter half year [36] (Fig 1). To the west of this region is a winter rainfall zone (WRZ) where more than 60% of precipitation occurs during the winter months. This rainfall pattern is influenced by the seasonal migrations of the westerlies which transport moisture to the WRZ during the austral winter months [37–38]. The southern westerlies are high altitude winds that blow from the west and define areas where cool dry air from the Antarctic collides with warm, moist air from the tropics causing instability and convection, resulting in rising moist air that creates cloud formation and precipitation [7]. To the east of the YRZ is the summer rainfall zone (SRZ) where tropical easterly winds bring moisture from the Indian Ocean during summers, while the winter months are arid with less than 30% rainfall [37–38] (Fig 1). It is likely that the extent of these rainfall zones may have shifted in the past [35, 37]. Current mean annual precipitation (MAP) at BBC is c. 500–600 mm [31, 39]. At present temperatures range from a mean daily summer maximum of 22°C, and in winter 12.6°C, with an average of 17.25°C for the whole year.

Currently there are three main biomes in the region known as the Hessequa Municipality (5734 km^2^) that is flanked by the lower Breede River to the west and the Gourits River to the east. These main biomes are fynbos (90.23%), succulent Karoo (8.61%) and Albany thicket (1.13%) [39–40]. However the BBC area is mainly associated with fynbos and thicket. In a 10 km radius of the site, Blombos strandveld and Albertinia sand fynbos dominate the landscape. Additionally there are pockets of Cape seashore vegetation and southern coastal forest [39–40]. The Blombos strandveld contains a mosaic of thicket and fynbos, where thicket genera include *Euclea*, *Olea*, *Cassine* and *Sideroxylon*; while prominent fynbos genera are *Phylica*, *Agathosma*, *Metalasia* and *Ischyrolepisas* [41]. The Albertinia sand fynbos is characterised by medium tall (1.5–2 m) open shrub, together with a denser layer of lower shrubs, and a ground cover of hemicryptophytes [39–40]. The vegetation is mainly comprised of Proteaceae (bushes and shrubs), though plants of the Restionaceae (reeds) family are wide-spread in moister environments such as on coastal edges and along watercourses [39–40]. The southern coastal forest is dense, low- to medium height, with a simple canopy [41]. It is represented by species such as *Sideroxylon inerme* (milk wood) growing on recent and Cenozoic coastal dunes, at the foot of deep river valleys, on fire-protected walls and at the bottoms of ravines [41]. The Cape seashore vegetation is usually comprised of open grassy and herbaceous cover, though the vegetation can also contain low-growing shrubs [40]. It is often dominated by a single pioneering plant species, the grass *Ehrharta villosa* var. *maxima*, and succulent shrub *Tetragonia decumbens*, are examples of pioneers that stabilise young dunes so that other plant species can become established as the dune matures [40]. Furthermore, the landscape in the immediate vicinity of BBC also comprises numerous rocky outcrops and patches of loose sandy soil with little or no vegetation cover.

Fresh water is available from springs 300 m and 600 m to the east of the site [31]. Along the south-western Cape coast fresh water springs are numerous and originate at the interface of the Table Mountain Group sandstones and the upper tidal reaches. The springs are fed by deep inland aquifers in the Bredasdorp Group and provide, apart from occasional rivers, one of the few dependable sources of water on the coastal plain between the Cape Folded Belt Mountains and the Indian Ocean [31]. It is likely that these sources of fresh water were attractive to both humans and animals in the MSA.

### Stratigraphy and dating

**Fig 3:**
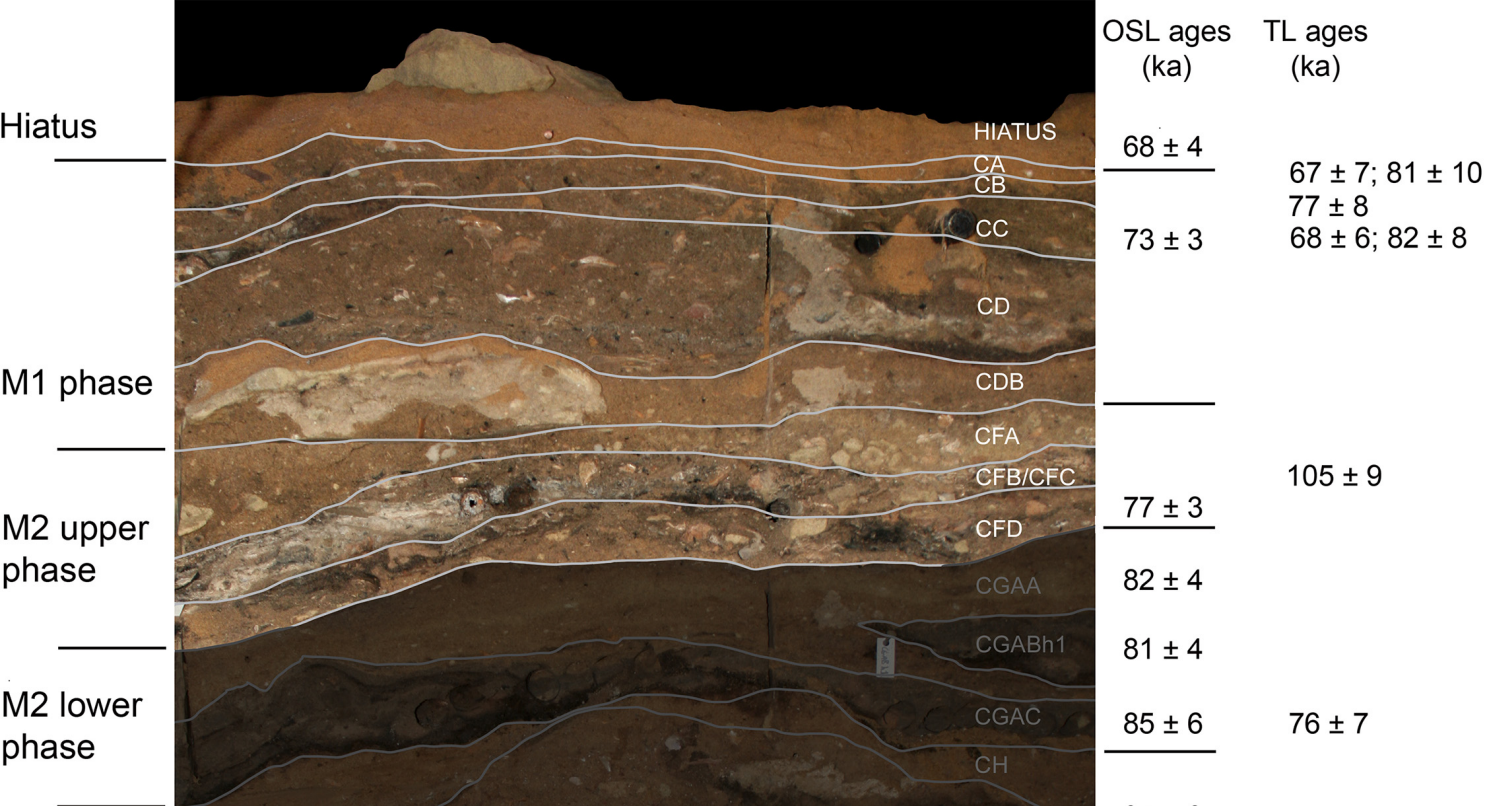
Stratigraphy, phases and dating from the South section of the BBC SB sequence.

The MSA levels at BBC are divided into four phases: M1, M2 Upper, M2 Lower, and M3. [11, 42–46]. In this paper we present the results of the analyses of micromammal remains and their palaeoecological implications from the M1 and M2 Upper phases associated with the Still Bay industry (Fig 3). The SB stratigraphy is characterised by clearly defined levels. Ground waters rich in calcium carbonate (CaCo_3_) have percolated through the cave roof and walls, creating a suitable environment for preservation of organic material, especially in proximity to hearths and ash deposits [31].

The SB levels have been dated by thermoluminescence (TL), optically stimulated luminescence (OSL) and electron spin resonance (ESR) [11, 13, 42–48] (Fig 3 & Table 1). There is an archeologically sterile dune sand level dated by OSL to 69 ± 5 ka and 70 ± 5 ka (Fig 3) [13, 42–45, 48], separating the final MSA levels from the oldest LSA level (c. 2 ka), documenting a major episode of cave mouth closure [31].

The M1 phase has been dated by OSL to 72.7 ± 3.1, 72.5 ± 4.6, 74.6 ± 3.9 [42–43, 48] and TL ages are 74 ± 5 and 78 ± 6 ka [46]. The M2 Upper phase also contains SB points, although in lesser quantities than in M1, and the OSL age is 76 ± 3 ka [45]. In 2010 the SB sequence was resampled for OSL dating, with new dates of c. 76–72 ka [48]. Taking into account the earlier dating results and those obtained using the TL method, we suggest that 76 ka should be regarded as the *terminus post quem* for the SB sequence at BBC.

The material culture, subsistence and palaeoenvironmental information characteristic of the M1 and M2 Upper phases are summarised in Table 2.

**Table 2:** Synopsis of the BBC material culture, subsistence and palaeoenvironmental information from the M1 and M2 Upper phases.

*Phase*	*Characteristics*	*Reference*
**Hiatus**	**Palaeoenvironment:** The culturally sterile layer formed during a period of sea level regression during the onset of global cooling (MIS 4)	[49]
**M1**	**Material culture:** Still Bay typological lithics—pressure-flaked bifacial foliate points made mainly from heat-treated silcrete	[17, 50–53]
	Shell beads—perforated *Nassarius kraussianus* tick shells with wear facets, some beads also have ochre staining or deliberate heating	[14–15, 54–56]
	Worked bone tools–formal and informal, polished points	[16, 57–59]
	Engraved ochres–abstract patterns	[13,15]
	**Subsistence:** Medium to high density deposits in CA-CDA. CDB low intensity. Small basin-shaped ash and carbon hearths.	[60]
	Large ungulates present, reduction in small browsers such as grysbok/steenbok (*Raphicerus* spp.)	[17, 19, 60–61]
	Shellfish and fish, although lower yields of shellfish in the upper layers of the phase	[17,62]
	**Palaeoenvironment**: Sand burrowing mussel (*Donax serra)* indicating a sandy beach within foraging range	[31]
	Reduction in small browsers may indicate more open environments thus a reduced habitat for these species	[60]
	Less shellfish in the upper layers of M1 indicate retreating sea levels	[17]
**M2 Upper**	**Material culture**: Same as in M1	See above
	**Subsistence**: CFA low intensity deposit, CFB and CFC/CFD medium to high intensity, large hearths	[60]
	Small ungulates such as grysbok/steenbok (Raphicerus spp.) common, in greater abundances than M1.	[17, 31, 61, 63]
	**Palaeoenvironment**: Intense shellfish exploitation in the lower SB deposits indicates the shoreline coming closer to the site during the first part of MIS 5a	[31]
	Water dependent southern reedbuck (*Redunca arundinum*) indicator for relatively wet conditions	[17, 60, 64]

## Material and Methods

In this paper we present the results of the analysis of small mammal remains recovered from layers CA-CFA during the 2000–2010 excavations at BBC (Fig 2). Permits for the excavation were obtained from the National Monuments Council (permits granted before 2000) and Heritage Western Cape, the Provincial Heritage Agency based in Cape Town, South Africa. The research permits to conduct archaeological excavations at BBC are issued under the National Heritage Resources Act (Act 25 of 1999) and the Western Cape Provincial Gazette 6061, Notice 298 of 2003. Excavation permits No. 8/96/06/001/51, 2003/12-001, 2005/05-005, 2007/03-003 and 2010/02-001 have been issued to CSH.

The micromammal remains are curated by Iziko Museums of South Africa, 25 Queen Victoria Street, Cape Town, 8001, and at the University of the Witwatersrand Satellite Laboratory, Buitenkant Street, Cape Town, Gardens, 8001, Cape Town. The micromammal assemblages are catalogued under the labels: BBC 2000, BBC 2002, BBC 2004, BBC 2005, BBC 2007, BBC 2008, BBC 2009 and BBC 2010.

In total 6150 micromammal elements from all excavated sub-quadrants of the M1 (CA-CD) and M2 Upper (CF-CFD) phases were analysed and catalogued. For the M1 phase, the micromammal data from CA to CCC were combined due to small sample sizes in these layers. The micromammal material was extracted from fragments larger than 1.5 mm (mesh size of sieve). Following the initial extraction, post-cranial and cranial elements were observed with a 40x Labomed Digizoom binocular light microscope. All necessary permits were obtained for the described study, which complied with all relevant regulations.

### Taphonomy

The suitability of the micromammal bones for palaeoenvironmental analysis was assessed by establishing the chemical and physical forces that may have affected and possibly biased the assemblage. Taphonomic aspects such as bone frequency, fragmentation and physical damage to the bones were quantified following the methodology developed by Andrews [65] and Fernandez-Jalvo & Andrews [66]. These analyses are essential to identify the predator(s) or agent(s) responsible for the accumulation of the micromammal assemblage and determine any potential bias [65–73].

Four main categories of taphonomic information were investigated; digestion (grade and frequency), representation of skeletal elements, breakage of skeletal elements, and physical post-depositional damage to the elements. Breakage patterns of crania and post-crania, skeletal element abundance, and the frequency and intensity of digestion were taken into consideration when identifying the predator species. The identification of predator(s) followed the list of categories as developed by Andrews [65]. Minor changes were done to the digestion classification, following the protocols of previous research on micromammals in South Africa [72, 74–76].

The effects of digestion are noticeable on the enamel of micromammal teeth [65]. Rodentia incisors are resistant to post-depositional breakage and are similar in shape and form. They have a large enamel and dentine surface which makes them ideal for analyses of predator-related digestion. Their shape is relatively uniform, making them suitable for comparison between taxa as opposed to molars [74]. In this study Rodentia incisors, both *in situ* and isolated, humeri and femora were microscopically analysed for predator-related digestive marks.

The skeletal element abundance was calculated following Andrews [65]: R*i* = N*i*/ (MNI x E*i*) where R*i* is the relative abundance of the element *i*, N*i* is the number of elements *i* in the assemblage, MNI is the minimum number of individuals and E*i* is the number of elements *i* in the prey skeleton. The calculation is based on the notion that the predator consumes most or all of the body of the prey and modern samples indicate that predators have characteristic patterns for the proportional abundances of prey elements [65].

Furthermore, taphonomic analyses established the processes which affected the BBC micromammals since their deposition. The surfaces of the osseous remains were investigated for post-depositional alterations caused by factors such as weathering, sediment-related corrosion, transport, trampling and etching from roots etc. [65–66, 73].

### Taxonomy

Taxonomic identification of the micromammal species was based on mandibles, maxillae and dental morphology following general practice (i.e. [69, 73, 77]). The identification was done using certain predefined frequent elements of maxillae and mandibles that vary according to order or genus. Mandibles and maxillae without teeth *in situ* were identified from alveoli characteristics. If the mandibles and maxillae could not be identified to species, they were assigned to either family or if possible sub-family. The taxonomic identification was aided by the Iziko South African Museums’ extensive collection of comparative specimens. Micromammal species from Klasies River main site and Boomplaas Cave (Fig 1), which had previously been identified by D. M. Avery, were used as reference, in addition to identification keys [78–79]. The post-cranial elements were identified by general assignation unless the element could be identified to order/family in the case of animals such as shrews, moles, mole rats or bats. The systematic classification used in the study followed the revised systematic checklist of Wilson and Reeder [80].

### Biodiversity

The micromammal assemblage was subject to estimates of biodiversity by using various indices, such as species richness, general diversity and evenness of the assemblage and the palaeoenvironmental information these indices provide. The results of the general diversity and dominance indices were subject to a *t*-test to estimate any potential statistical significance across the stratigraphical layers. The distribution of taxonomic composition through time was analysed by presence-absence and relative abundance of taxa and a taxonomic habitat index (THI). All statistical calculations, unless otherwise stated, were done using the free software program for data analysis; Paleontological Statistics (PAST) [81].

The number of taxa (richness) and the number of individuals per species (diversity) serves as a measure of vegetation structure. Simple vegetation structures dominated by a small number of plant species have correspondingly low micromammal species richness and diversity [65, 82]. Thus the number of micromammal species is proportional to vegetation structure, i.e. complex vegetation structures have greater biodiversity [83].

Precipitation has been suggested as a reliable proxy for environmental productivity [84]. Andrews and O’Brien [85] noted a correlation between *small mammal* species richness and maximum monthly precipitation (MMP). Furthermore, the results of their large scale analysis of mammal species diversity in southern Africa suggest that small mammal species richness correlates with seasonal distribution of rainfall [85]. The diversity of species is greater in areas where the seasonal variation in precipitation is moderate. In general small mammal richness is best described as a function of decreases in seasonal variability in the thermal, energy and precipitation regimes. Thus small mammal species richness is expected to be greater where fluctuations in these climate variables are limited [85–87].

The Shannon Wiener index, H, was used to evaluate the general diversity of the micromammal population; H = - Σ *P*_*i*_ (ln *P*_*i*_) where *P*_*i*_ is the proportion (P) of taxon *i* in the assemblage. Taxonomic evenness was calculated as E = H/lnS where S is taxonomic richness. The Simpson index indicates the probability that two randomly picked individuals are of the same species [88]. The Simpsons index of dominance, D, is given as D = Σ (p^2^_*i*_) where p_*i*_ = *n*_*i*_/*n* (the proportion of species *i*).

The taxonomic composition was compared by applying two similarity indices to evaluate the assemblage on a high level (presence-absence) and low level (proportional abundance). The Jaccard index was based on identified taxa and binary data, expressed in a dendrogram as unconstrained paired groups. The Bray-Curtis similarity index is based on distance measure which is converted by subtracting from one to obtain similarity indices [88]. The relative abundance of each species was calculated and expressed as a percentage representation for each stratigraphic layer. This was done to avoid results influenced by sample size. Exclusion of grouped taxa was done for both indices as these categories are most likely a result of fragmentation of identifiable elements which hampered exact identification, and could potentially create an artificial variation based on fragmentation.

Taxonomic habitat index (THI) is a cumulative index obtained by combining the habitat indications of all species contained in an assemblage [65, 89]. The ecological preference of the taxa is based on the habitat in which the extant species live [65, 73]. For each taxon, a score is allocated to various types of pre-established vegetation in which the species can be found, the sum of these scores always being equal to 1. We used available literature on the species ecological requirements and distribution to allocate the scores with the greatest possible accuracy. In essence the THI is a method of aggregating the information of each micromammal taxa present in a layer into a composite interpretation of the palaeoenvironment. When the values for all species are added together the cumulative index may indicate the dominant habitats in the area [65, 73, 90].

Through THI local expansion or retreat of habitat types, complete disappearance of a given type of habitat, appearance of new habitat types, and/or no changes or homogeneity of habitat composition through time are assessed [83]. The index can also provide information on general climatic conditions. Optimal conditions would result in a complex vegetation structure while a simple vegetation structure, indicated by dominance of one habitat type, would suggest stressed environmental conditions [83].

The distribution, preferred habitats and habits of the micromammal taxa, used to derive palaeoenvironmental information and the taxonomic habitat indices, were based on Skinner and Chimimba [91], with supplementary information from Avery [72, 77–78, 92–97], Avery *et al*. [36], Bigalke [98], Bond *et al*. [99], Davis [100], De Graaff [79], Delany [101], Fleming and Nicolson [102], Hopley *et al*. [103], Matthews [74], Matthews *et al*. [73, 75–76], Meester [104], Meester *et al*. [105], Perrin [106–108], Rautenbach [109], Roberts [110], Schraden and Pillay [111], Shortridge [112–113], Stuart and Stuart [114] and Wilson and Reeder [80].

## Results and Discussion

### Taphonomy

#### Establishing the predator

**Fig 4:**
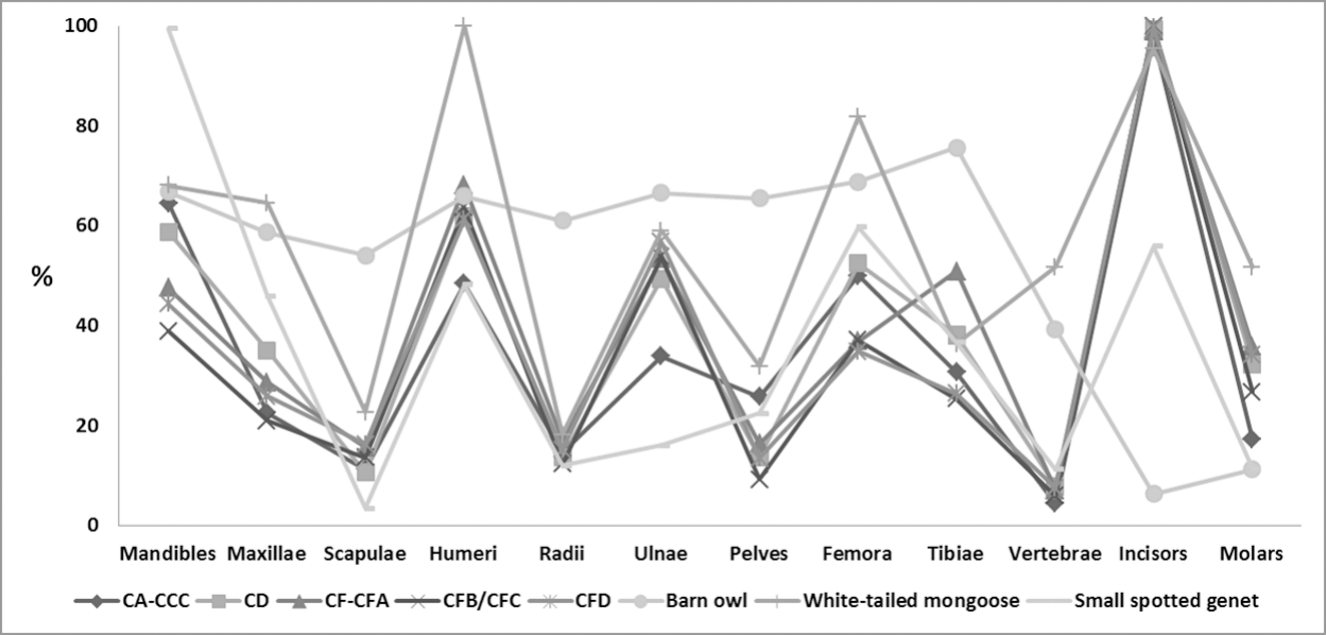
Skeletal element abundance for the SB sequence at BBC. Examples of modern skeletal element abundances with similar compositions are based on Andrews [65].

Breakage of post-cranial and cranial elements was extensive throughout the SB levels (Table 3, Fig 4) (see also Nel [115]). Both limb bone fragmentation and average relative abundance indicate extensive breakage and loss of skeletal elements (Table 3). The skeletal element abundance (SEA) for M1 and M2 Upper (Fig 4) were compared with carnivores that typically predate on micromammals. All layers showed a similar SEA pattern as mammalian carnivore predators; i.e. small-spotted genet (*Genetta genetta*) or mongoose species [65] (Fig 4). This is strongly in opposition to the digestive etching seen on incisors, femora and humeri in the assemblage (see discussion below). The result highlights a common problem when analyzing micromammal assemblages from archaeological sites [65, 74]. There are a number of post-depositional damage types which could influence and bias the SEA pattern, and the SEA method seems more useful in identifying a predator based on pristine pellet and scat assemblages [65]. Throughout the SB sequence there are several occurrences of large and medium sized limestone blocks spalling from the cave roof onto deposits, thus some breakage can be attributed to these events in addition to trampling of sediments by human occupants. Consequently breakage indices and SEA produced ambiguous results and were found not suitable for assessing the type of predator at BBC.

**Table 3:** Recorded breakage of humeri in the M1 and M2 Upper phase.

Phase	Layer	Distal	Distal+Shaft	Proximal	Proximal+Shaft	Shaft	Complete	Total N	ARA	NSP
**M1**	**CA-CCC**	43.3	13.3	16.7	0.0	0.0	26.7	30	21.7	611
	**CD**	50.5	11.1	28.3	4.0	0.0	6.1	99	26.2	2109
**M2 Upper**	**CF-CFA**	48.2	10.8	26.5	3.6	0.0	10.8	83	26.3	1623
	**CFB/CFC**	64.1	3.9	23.3	1.9	1.0	5.8	103	21.2	1781
	**CFD**	59.6	11.0	20.2	1.8	0.0	7.3	109	22.4	2201

Breakage is expressed in percentage where N is the total number present in the layer. The average relative abundance (ARA) is the means of the relative abundances for all skeletal elements, except for the isolated teeth, in each layer. If there was no loss of skeletal elements the average relative abundance should be 100%, as there would be no loss from the cranial and post-cranial skeleton.

All layers in the M1 and M2 Upper phases have similar digestion patterns and frequencies on the incisors, humeri and femora (Table 4). The majority of the recorded digestion is classified as light. In southern Africa, predator species associated with light digestion are the African barn owl (*Tyto alba affinis*), African grass owl (*Tyto capensis*) and marsh owl (*Asio capensis*) [96]. African grass owl and marsh owl nest, hunt and roost in open grassland while African barn owls use rocky ledges or caves for roosting [116]. The African barn owl utilises a wider range of terrain for hunting, thus given the diversity of taxa present (Table 3) it is possible that this species was responsible for bringing most of the micromammals to the cave. As neither grass owls nor marsh owls return on a regular basis to the same roosting site, this further supports the idea that barn owls were the primary accumulators [117]. African barn owls are opportunistic predators, preying on the most abundant micromammal taxa present. As a result, the variation of species composition in a micromammal assemblage, where the African barn owl is the assumed predator, is likely to reflect the taxonomic diversity at the time and not significantly bias the assemblage.

**Table 4:** Percentage representation of digestion on incisors (combined *in situ* and isolated), femora and humeri from the SB sequence at BBC.

*Phase*	*M1*		*M2 Upper*		
**Digestion %**	**CA-CCC**	**CD**	**CF-CFA**	**CFB/CFC**	**CFD**
**Incisors**					
**No digestion**	62.9	59.1	59.7	54.9	58.5
**Light**	28.8	32.6	31.4	35.0	30.4
**Moderate**	5.3	6.8	6.5	5.9	5.4
**Heavy**	3.0	1.0	2.0	2.4	2.3
**Extreme**	0.0	0.4	0.3	1.7	0.8
**Total digested**	37.1	40.5	39.9	43.4	40.8
*N*	*132*	*279*	*293*	*286*	*260*
**Humeri**					
**No digestion**	36.7	45.5	35.4	45.6	45.0
**Digested**	63.3	54.5	64.6	54.4	55.0
*N*	*30*	*99*	*82*	*103*	*109*
**Femora**					
**No digestion**	41.9	31.0	6.7	35.0	16.4
**Digested**	58.1	69.0	93.3	65.0	83.6
*N*	*31*	*84*	*45*	*60*	*61*

The division of incisor digestion classes is based on Andrews [65].

**Fig 5:**
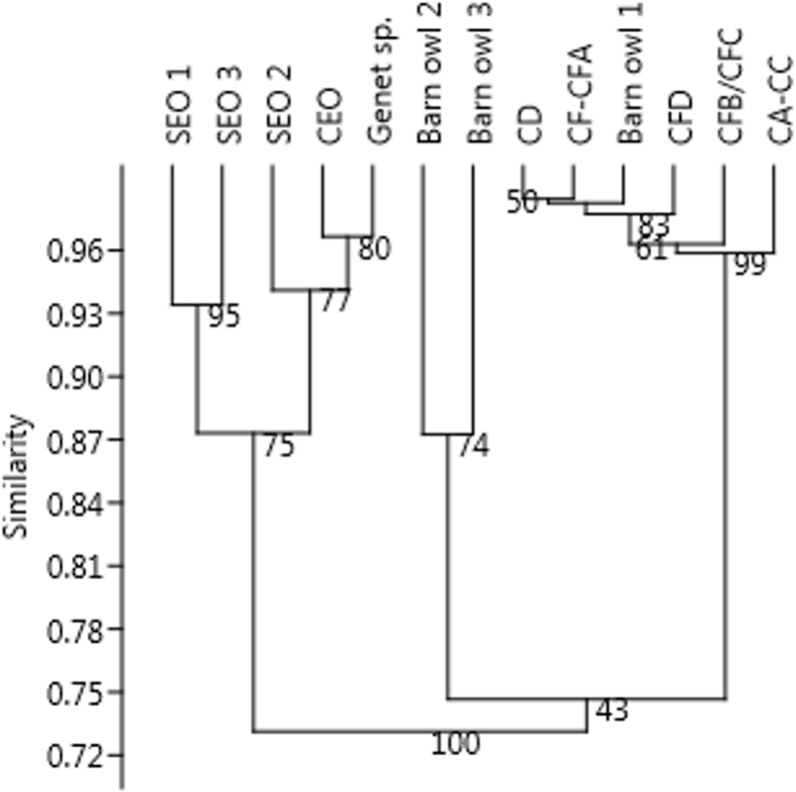
Bray-Curtis index showing similarity distances based on digestion of incisors from modern samples and the BBC micromammal samples from the M1 and M2 Upper phases. The results are presented as a dendrogram with unconstrained pair groups and have been bootstrapped (n = 9999). SEO = spotted eagle owl (*Bubo africanus*), CEO = Cape eagle owl (*Bubo capensis capensis*). Values of modern samples are based on Nel [115].

The Bray-Curtis similarity index shows that the digestion traces on the micromammal samples in M1 and M2 Upper are more similar to the barn owl modern samples than the other modern predators represented (Fig 5).

#### Post-depositional modification

**Fig 6:**
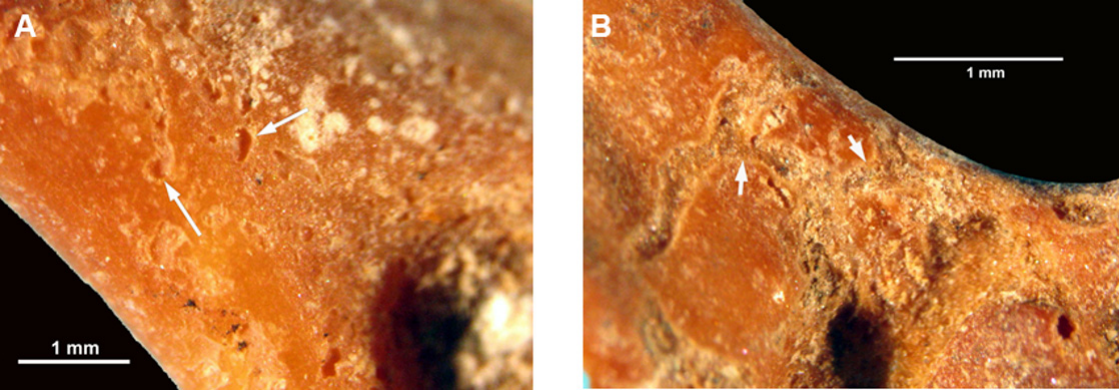
Examples of the most frequent post-depositional damage on the elements in the M1 and M2 Upper phases. (A) Pitting of humerus and (B) etching of humerus (both from layer CC, sub-quadrant E5b).

Post-depositional modifications to the surface of the bones indicate several mechanical issues affecting the micromammal remains after deposition. Etching and pitting of elements occurs throughout the SB sequence (Fig 6). Etching is particularly more frequent in high density deposits such as the SB layers and is probably due to the increased acidity of the sediments caused by the decomposition of organic materials [118]. High density deposits by human occupants, which contained for example plant material, faecal matter, unutilised animal remains etc., could then raise the acidity of the soil in the specific areas of the site.

Rounded breaks are present on digested and undigested elements and this is probably due to post-depositional mechanical action, for example sand abrasion. This damage is particularly prevalent in the M1 phase. Cemented sand stuck to the surface of the bones, and when this matrix was removed, they appeared smooth and polished. This is particularly the case for elements in layer CDB in sub-quadrants F6d, G6c, G6d and H6c. These quadrants are located near to the entrance of the cave where one could expect increased trampling (Fig 2). Other damage such as corrosion and desquamation were limited, indicating that weathering did not affect the assemblage to any degree. Burnt elements were not frequent in M1 and M2 Upper, and were probably burnt ‘accidentally’ while *in situ* [115]. Voorhies’ categories [119–120] of fluvial transport indicate that water transportation did not affect the micromammal assemblage [115].

### Biodiversity–palaeoecological implications

#### Diversity indices

**Fig 7:**
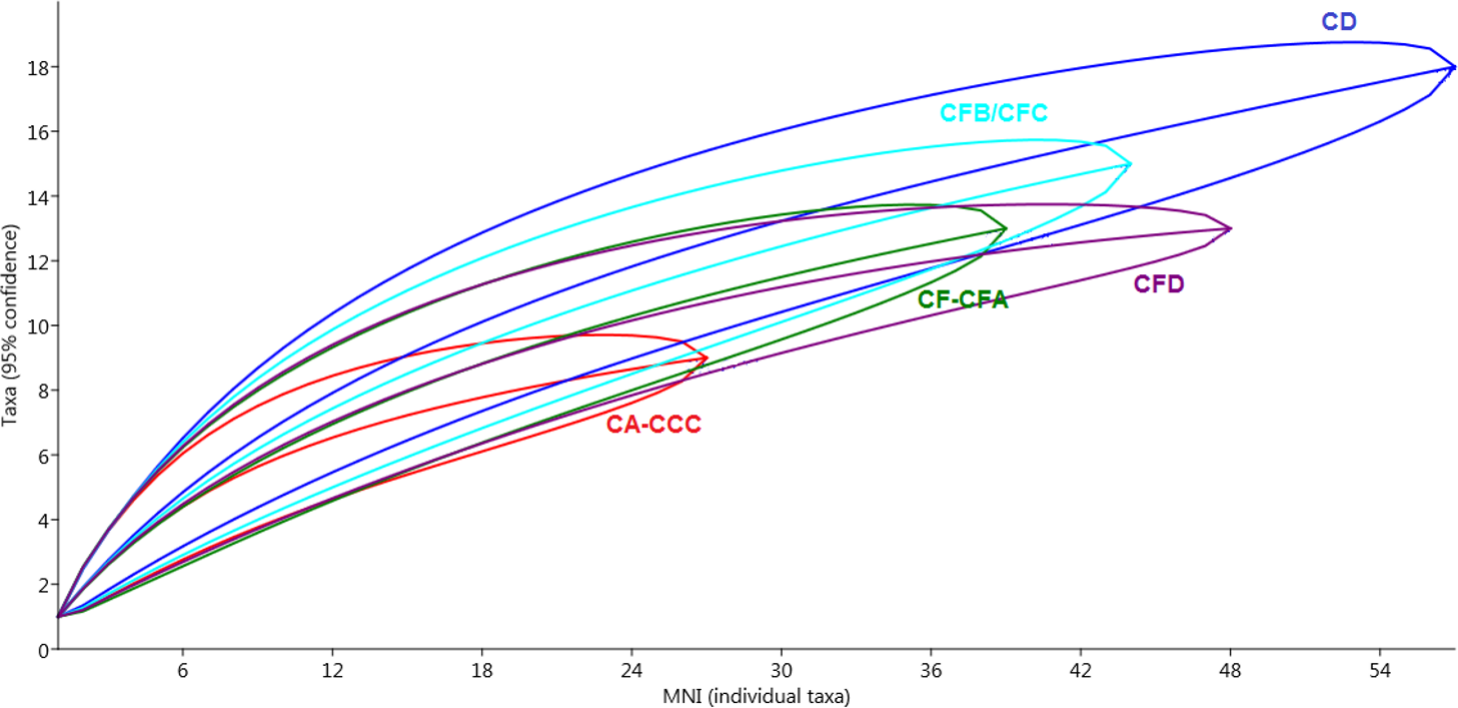
Point by point individual rarefaction curves for M1 and M2 Upper layers at BBC with estimated species richness and 95% confidence interval.

The number of taxa (richness) and the number of individuals per species (diversity) can provide general information of habitats, but both estimates are sensitive to sample size [86, 88]. To correct for this rarefaction analyses were applied to investigate the effects of sample size upon taxon counts in the various layers (Fig 7, see S1 Table: Standardisation of individual rarefaction curves).

To examine the apparent discrepancy and similarity between the layers, the smallest sample (CA-CCC) was standardised, followed by rarefaction of the other samples to the set standard size. The rarefied standardised taxon count was compared statistically using a *t* test (p < 0.05) based on the equality of means. The variance between layers CA-CCC and CD was statistically significant (t = -2.16, p<0.035) (S1 Table: Standardisation of individual rarefaction curves). The other layers had statistically insignificant values compared to the standardised sample. These results were confirmed by permutation tests. The variation between CA-CCC and CD in the M1 phase indicates that there were changes in environmental productivity which led to a decline in species richness from layers CD to CA-CCC, as the number of species present in a layer is proportional to vegetation structure [83]. Following Andrews and O’Brien’s [85] correlation of small mammal species richness and seasonal variation in precipitation for small mammals, the decline observed from CD to CA-CCC suggests a seasonal variation in precipitation in MIS 5a/4.

Diversity, calculated here by the Shannon-Wiener index, *H*, has been used as a measure of environmental stability in palaeoenvironmental reconstruction. The index takes into account both the number of taxa present, and the relative abundance (evenness of representation) of each taxon [88]. Avery [77, 97, 121] has demonstrated *H* to co-vary with known climatic conditions.

**Fig 8:**
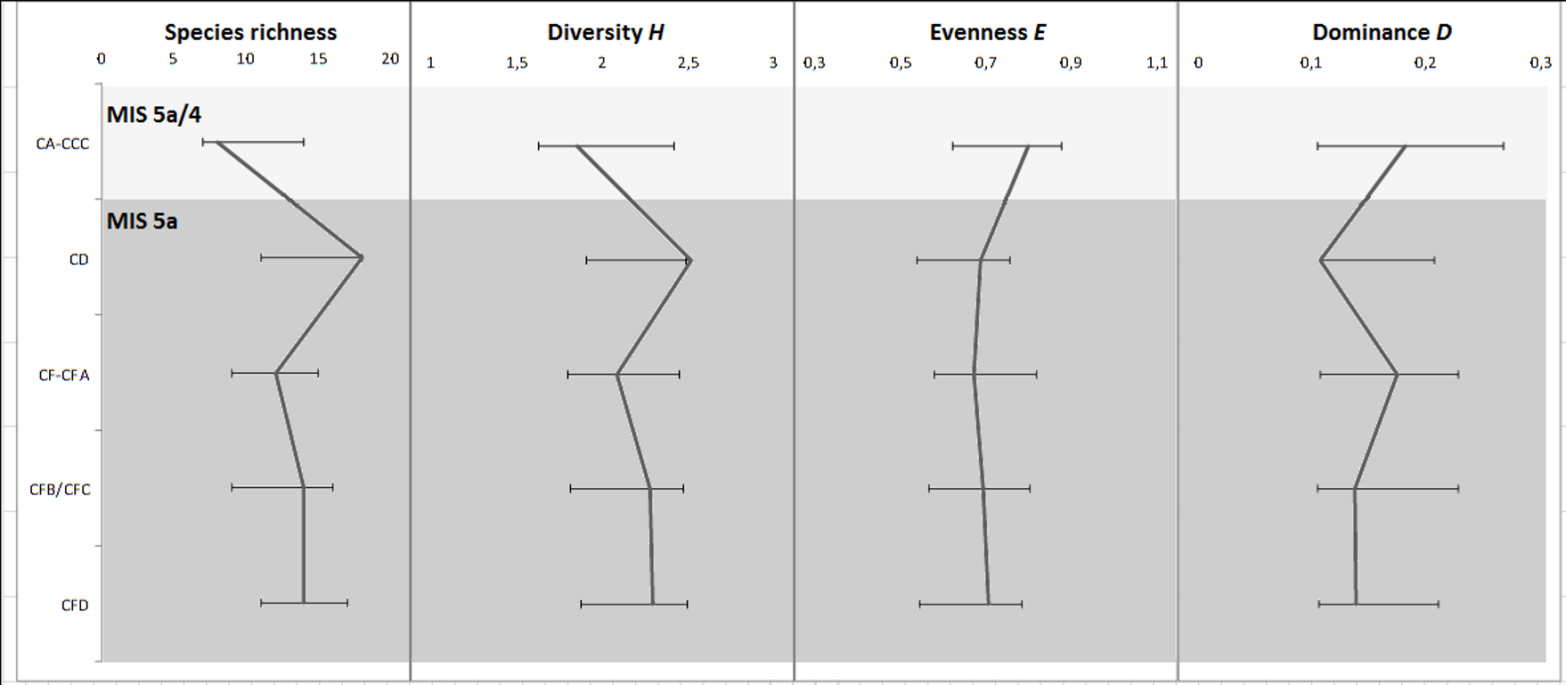
Diversity indices for the layers of the Still Bay sequence at BBC. Species richness, diversity *H*, evenness *E* and dominance *D* are expressed with 95% confidence intervals.

Layers CA-CCC have the least diverse assemblage where *H* = 1.85. This was expected as the index is dependent on relative frequencies and species richness [88, 122] and layers CA-CCC have the smallest sample with fewer taxa present than in other layers. The greatest diversity is seen in layer CD of the M1 phase where *H* = 2.51 (Fig 8). In general, the diversity indices throughout the SB sequence are high with the exception of CA-CCC. This suggests that the area around the cave supported a diverse micromammal community during MIS 5a, followed by a decline in diversity during the transition to MIS 4. Avery [87] has noted that *H* demonstrably rises during interglacial periods and declined during glacial periods. The diversity decrease in layers CA-CCC suggests this layer falls within the transition period leading to MIS 4.

Evenness, *E*, is calculated based on the Shannon-Wiener index and the same sensitivity with regards to relative abundance and number of taxa present (Fig 8). Simpsons index of dominance, *D*, is a better estimate of the composition of taxa as it is less affected by species richness which is linked to sample size. The greatest dominance is in CA-CCC with 0.18, while CD has the lowest dominance with 0.11. The relatively low dominance throughout the SB sequence spanning MIS 5a may suggest a reasonably varied species composition in these layers, again indicative of a complex vegetation structure. The higher numbers in layers CA-CCC indicate comparably less varied vegetation in MIS 5a/4.

The Shannon Wiener index for general diversity and the Simpson index for dominance were statistically compared for all layers by using a standard *t* test where *p*<0.05 (Table 5). The diversity indices in layers CA-CCC and CD were significantly different at p<0.05 (t = -2.9, p<0.005). The result indicates that there were significant changes in micromammal diversity (representation and relative proportions) in the vicinity of BBC during the MIS 5 to MIS 4 transition. Based on the diversity indices, BBC was an area with a relatively complex vegetation structure likely comprising a range of ecotones during MIS 5a, particularly towards the latter part of the stage in CD. A negative change in environmental conditions, that affected the diversity of micromammals, is likely during the transition from MIS 5a to MIS 4.

**Table 5:** *P* values of the t test based on the results of the Shannon Wiener and Simpson indices.

**Simpson index**	***p*(same)**	**Shannon Wiener index**				
	**Layer**	**CA-CCC**	**CD**	**CF-CFA**	**CFB/CFC**	**CFD**
	**CA-CCC**		***0*.*005***	0.421	0.080	0.232
	**CD**	0.178		0.072	0.323	0.091
	**CF-CFA**	0.914	0.199		0.410	0.776
	**CFB/CFC**	0.508	0.474	0.484		0.539
	**CFD**	0.795	0.259	0.731	0.680	

Significance at p<0.05 are in bold italic.

### Taxonomic composition and local environmental implications

The BBC micromammal assemblage from the SB sequence comprises a total of 263 minimum numbers of individuals (MNI) from 21 different taxa (Table 6). There is one species of Chrysochloridae, four taxa of Soricidae, one *Bathyergidae*, eleven species from the Muroidea superfamily, one *Gerbilliscus* and three species of Chiroptera.

**Table 6:** Stratigraphic distribution of the taxonomic representation in M1 and M2 Upper at BBC.

Phase	M1		M2 Upper		
	CA-CCC	CD	CF-CFA	CFB/ CFC	CFD
*Chlorotalpa duthieae*	0	0	0	0	1.7
*Crocidura flavescens*	0	1.4	2.0	1.9	1.7
*Myosorex varius*	3.3	12.5	12.0	15.4	13.6
*Crocidura cyanea*	3.3	0	0	0	0
*Suncus varilla*	0	2.8	8.0	7.7	10.2
*Georychus capensis*	0	1.4	0	0	0
*Myomyscus verreauxii*	0	4.2	4.0	3.9	3.4
*M*. *verreauxii/Mastomys sp*.	3.3	4.2	0	1.9	3.4
*Dendromus mesomelas*	0	1.4	2.0	0	3.4
*Dendromus melanotis*	6.7	1.4	2.0	1.9	0
*Dendromus sp*.	0	1.4	0	0	3.4
*Mus minutoides*	0	5.6	0	1.9	3.4
*Dendromus sp*.*/ M*. *Minutoides*	0	0	2.0	0	0
*Steatomys krebsii*	0	1.4	0	1.9	0
*Dasymys incomtus*	0	1.4	0	1.9	0
*Rhabdomys pumilio*	23.3	16.7	26.0	21.2	23.7
*Acomys subspinosus*	0	1.4	4.0	1.9	0
*Otomys irroratus*	6.7	5.6	6.0	5.8	5.1
*Otomys saundersiae*	3.3	1.4	0	1.9	0
*O*.*irroratus/O*.*saundersiae*	3.3	0	2.0	0	3.4
*Otomys sp*.	3.3	15.3	18.0	13.5	8.5
*Gerbilliscus afra*	0	1.4	2.0	3.9	1.7
*Rhinolophus capensis*	13.3	5.6	2.0	0	5.1
*Rhinolophus clivosus*	13.3	8.3	6.0	3.9	3.4
*Minopterus schreibersii*	16.7	5.6	2.0	9.6	5.1
**TOTAL**	100	100	100	100	100
**MNI**	30	72	50	52	59
**NSP**	**611**	**2109**	**1623**	**1781**	**2201**

The numbers are expressed as relative abundances.

#### Similarity indices

**Fig 9:**
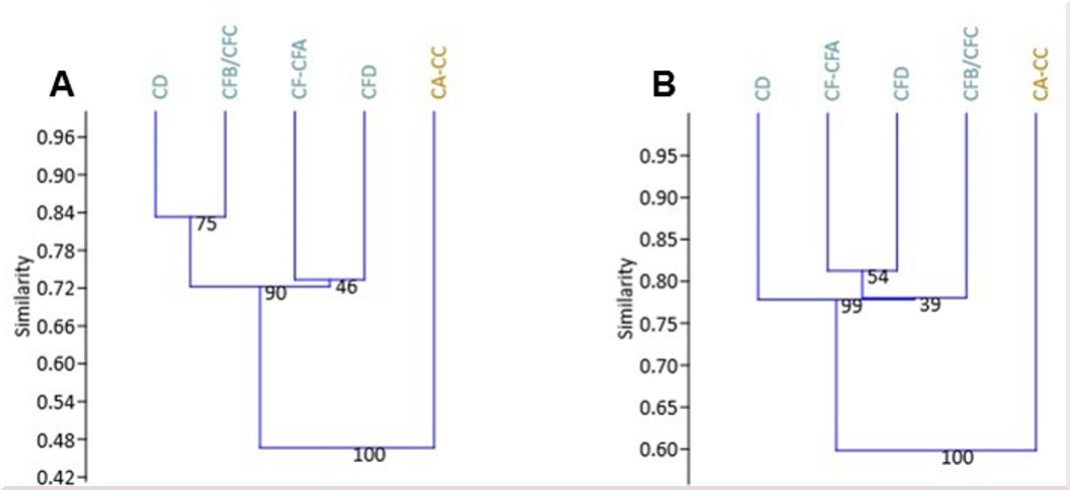
Similarity indices evaluating species composition. (A) Jaccard similarity is illustrated by unconstrained paired groups based on presence-absence of taxa. (B) Bray-Curtis similarity is illustrated by unconstrained paired groups based on relative abundance of taxa. The results were bootstrapped (n = 9999). Layers dating to MIS 5a are in turquoise and layers dating to MIS 5a/4 in gold.

The similarity of the taxonomic composition throughout the SB sequence was compared on a high rank level (presence/absence) (Fig 9A) and low level by relative abundance of species (Fig 9B). The MIS 5a layers have high levels of similarity (Fig 9). Meanwhile layers CA-CCC differ from the MIS 5a layers with regards to taxonomic composition and relative abundances (Fig 9), particularly on a high rank level where the similarity is 0.48. The result indicates less diverse vegetation structure in MIS 5a/4 compared to MIS 5a, which would affect the number of species present in the vicinity of BBC during that time.

#### Climatic variables

Detailed inferences of climatic variables based on taxonomic composition were difficult to assess as most of the taxa represented in the BBC micromammal sample have relatively wide tolerances to precipitation and temperature variables. This is comparable to other micromammal samples analysed from archaeological sites in the Western Cape (e.g. [73, 76]). The implications regarding general rainfall, seasonality and temperature thus remain tentative and should be viewed by taking into account palaeoenvironmental data from other regions.

Avery *et al*.’s [36] study of modern African barn owl pellets from sites in the Western Cape, suggested that high winter rainfall encouraged breeding activity in *Otomys irroratus*. A maximum-entropy approach for species habitat modelling supports the idea that *O*. *irroratus* actually prefers equal rainfall distribution throughout the year, for example the conditions associated with fynbos and Albany thicket biomes [123]. The continuous presence of *O*. *irroratus* in the M2 Upper and M1 phases (Table 3) indicates that the rainfall distribution was probably appropriate for the species requirements.

*O*. *irroratus’* relative abundance could also suggest that the amount of winter rainfall during M2 Upper and M1 phases did not increase, given that the species’ abundance remained relatively consistent throughout these phases (Table 3). However, due to the fragmentation of micromammal bones taxonomic identification of the Otomyinae was reduced (in particular *O*. *irroratus* and *Otomys saundersiae*), and it is thus not possible to confirm that *O*. *irroratus* abundances were, in fact, equal throughout the period (Table 3: Otomyinae 17.0% CFD, 21.2% CFB/CFC, 26.0% CF-CFA, 22.3% CD and 16.1% CA-CCC).

*Gerbilliscus afra* is endemic to the Western Cape. Their breeding is seasonal and confined to the post winter rainfall period [36, 91]. Avery *et al*. [36] cautiously indicate that the species current distribution, based on modern samples from barn owl pellets, is associated with near-coastal sites in the south and west of the Western Cape where winter rainfall is at least 59%. *G*. *afra* has low relative abundances in the BBC assemblage, perhaps indicating that the climatic conditions or their habitat requirements were on the verge of the species tolerance.

*Rhabdomys pumilio* at BBC likely belongs to a coastal clade of the species associated with fynbos and the succulent Karoo biome [124]. According to du Toit *et al*.*’s* [124] ecological niche model, altitude is an important variable limiting *R*. *pumilio* distribution in the Western Cape, while mean annual temperature and rainfall seasonality are playing a secondary, but significant, role [124]. The coastal clade prefers relatively higher mean annual temperatures found in lowland areas, and when temperatures increase beyond a certain point the predicted suitability of a given habitat declines rapidly. The niche model indicates that highest predicted presence of *R*. *pumilio* is mostly confined to winter rainfall areas, and their occurrence probability declines with increased rainfall outside of the winter season [124]. Due to the shallow off-shore topography (Fig 1), the altitude of BBC would not have changed significantly, even when sea levels declined, and is therefore not a significant factor. The decrease in *R*. *pumilio* abundance in CD could indicate a short period of either more aseasonal rainfall and/or changes in mean annual temperatures. This is tentative and should be seen in correlation with indications of possible changes in the vegetation substrate which may also have affected their abundance.

The latter part of MIS 5 and MIS 4 could be associated with increased seasonal rainfall in the Western Cape and along the southern coast in the YRZ [38, 93, 125]. *Myosorex varius* breed during warm, moist summer months [91, 114]. This suggests conditions suitable for breeding occurred during the SB period with a possible exception in the upper layers, CA-CCC (Table 3). *Suncus varilla*, also a seasonal breeder [91], is present in the M2 Upper phase, but decline in CD and are absent from CA-CCC (Table 3). The overall decline of Soricidae (Table 3) as the SB progressed suggests that the summer seasons were colder approaching the onset of MIS 4 and that there was possibly increased seasonal winter rainfall with lower mean annual temperatures (MAT).

#### Vegetation and substrate

Golden moles (*Amblysomus hottentotus*, *Chlorotalpa duthieae*, *Chrysochloris asiatica*) are present in the lower phases at BBC that are older than 77 ka and associated with MIS 5c-5b [115]. They are no longer present after CFD in the M2 Upper phase. Their preferred vegetation varies but all species needs loose sandy soil for burrowing. Their absence in the upper layers of the M2 Upper phase and M1 phase suggests less sandy soil availability in MIS 5a and towards the transition to MIS 4 [115]. The low numbers of *G*. *afra*, also a burrower in loose soil [91], supports this implication (Table 3). Expanding herbaceous and grass cover in MIS 5a would lead to more compact soil. The presence of large mammal grazers such as black wildebeest (*Connochaetes gnou*) and hartebeest (*Alcelaphus buselaphus*) in the M2 Upper, and particularly in the M1 phase, supports a higher grass component in the M2 Upper and M1 phases [17, 19, 60, 63].

The species of micromammals present confirms the presence of fynbos throughout the SB sequence but with a decline in the later SB period corresponding to layers CA-CCC. Ground *proteas* are dependent on *M*. *verreauxii* for pollination [73] and its occurrence indicates that fynbos vegetation was present during MIS 5a, with a decrease of ground *proteas* and fynbos during late MIS 5a/4. The presence of fynbos in MIS 5a is further supported by *Acomys subspinosus*, a fynbos endemic species, and *Chlorotalpa duthieae*, associated with dune fynbos [91, 126].

The presence of *Dendromus mesomelas* and *Dendromus melanotis* are associated with a decline in dense herbaceous cover at Klasies River main site [93] (Fig 1). These species are associated with tall grasses [91]. The increase in abundance of these species in layers CFB/CFC and layer CD suggests denser herbaceous cover during MIS 5a compared to the MIS 5a/4 transition. The area around the cave also consisted of open or less dense vegetation habitats during the SB, and this is supported by the ability of the African barn owl taking *M*. *minutoides*, a minute species of 2–12 g that is not easily spotted in dense vegetation [94].

#### Taxonomic anomalies

Chiroptera are particularly abundant in layers CA-CCC and their representation is 43.3% of the relative abundance of micromammals in these layers (Table 3). The bats *Minopterus schreibersii*, *Rhinolophus capensis* and *Rhinolophus clivosus* have communal roosting sites [127]. An increase in their numbers in the later SB could have enticed the African barn owl to prey on them, hence the greater numbers of digested post-cranial bat elements found in these upper layers. Presently, bats roost in a cave c. 20 m east of the BBC entrance. Bats are not favoured prey of the African barn owl though Avery *et al*. [36] records *M*. *schreibersii*, *R*. *capensis* and *R*. *clivosus* in modern pellets samples collected in the Western Cape Province. Elsewhere, Chiroptera have been recorded in relative abundance from modern barn owl assemblages in Bolivia where they accounted for 51% of the diet [128]. It is possible that an increase in bats as prey is due to a decrease in Soricidae and Otomyinae during the MIS 5a/4 transition (Table 3).

### Taxonomic habitat index

The present vegetation within a c. 10 km radius of BBC is Blombos strandveld, Albertinia sand fynbos, Cape seashore vegetation and southern coastal forest [39–40].The niche models developed for our Taxonomic Habitat Index (THI) focus specifically on vegetation microhabitat, and comprise categories that are based on the characteristics of the vegetation types currently present in the BBC area and the specific habitats of the extant micromammals in the study assemblage (Table 7).

**Table 7:** Taxonomic habitat index for the micromammal species present in the M1 and M2 Upper phases at BBC.

	Vegetation						
Species	Moist grass	Dry grass	Bush	Shrubland	Coastal scrub	Rocky	Sandy
*Chlorotalpa duthieae*			0.05	0.25			0.70
*Crocidura flavescens*	0.20	0.20	0.20	0.20		0.20	
*Myosorex varius*	0.30		0.10	0.30	0.30		
*Crocidura cyanea*	0.20	0.20	0.10	0.20	0.20	0.10	
*Suncus varilla*		0.80	0.20				
*Georychus capensis*		0.10					0.90
*Myomyscus verreauxii*	0.20	0.20	0.10	0.30	0.20		
*Dendromus mesomelas*	0.45		0.35		0.20		
*Dendromus melanotis*	0.45		0.35		0.20		
*Mus minutoides*	0.25	0.25	0.05	0.20	0.05	0.20	
*Steatomys krebsii*		0.33			0.33		0.34
*Dasymys incomtus*	1						
*Rhabdomys pumilio*	0.30	0.40	0.15		0.15		
*Acomys subspinosus*				0.80		0.20	
*Otomys irroratus*	0.40	0.40			0.20		
*Otomys saundersiae*		0.40			0.40	0.10	
*Gerbilliscus afra*							1

The THI consists of seven categories where five are descriptive of local vegetation (moist grass, dry grass, bush, shrubland, coastal scrub), while the remaining two categories describe substrate (rocky and sandy). The substrate components were included as they comprise habitats which are specific for some of the micromammal species recovered.

*Moist grass* category is associated with damp grass close to reed-beds, streams, vleis and dams, and is generally thick riverine grass characterised as dense vegetation. The grassy elements of the Restionaceae family in the Albertina sand fynbos are wide-spread in moist environments such as coastal edges and along watercourses.

*Dry grass* generally signifies open habitat with tall grasses and is similar to savanna type vegetation as well as the grassy dunes associated with Cape seashore vegetation. *Bush* is the coastal forest margins and low succulent vegetation, such as thicket elements in the Blombos strandveld, and includes dense vegetation growing on nutrient rich soil.

*Shrubland* includes shrubby low to medium height vegetation, and is particularly associated with the *Proteaceae spp*. and *Ericacea spp*. of the Albertinia sand fynbos and the Blombos strandveld. Shrubland includes low-growing woody elements and a dense, layered vegetation cover similar to heather. *Acomys subspinosus* is endemic to fynbos vegetation, feeding mainly on nutlets of *Restio spp*. [91] and therefore weighted 0.8 in this category but the species is also confined to rocky areas, thus 0.2 is assigned to this category.

*Coastal scrub* signifies patchy, sparse vegetation associated with succulent elements of the Cape seashore vegetation and other xeric ground-growing vegetation that occurs on nutrient poor soil and sandy substrates. The *rocky* component refers to hilly outcrops and slopes favoured by some micromammals. The *sandy* category is alluvium dry soil and sandy loams. The latter category may, in particular, refer to the likely vegetation in the BBC area.

**Fig 10:**
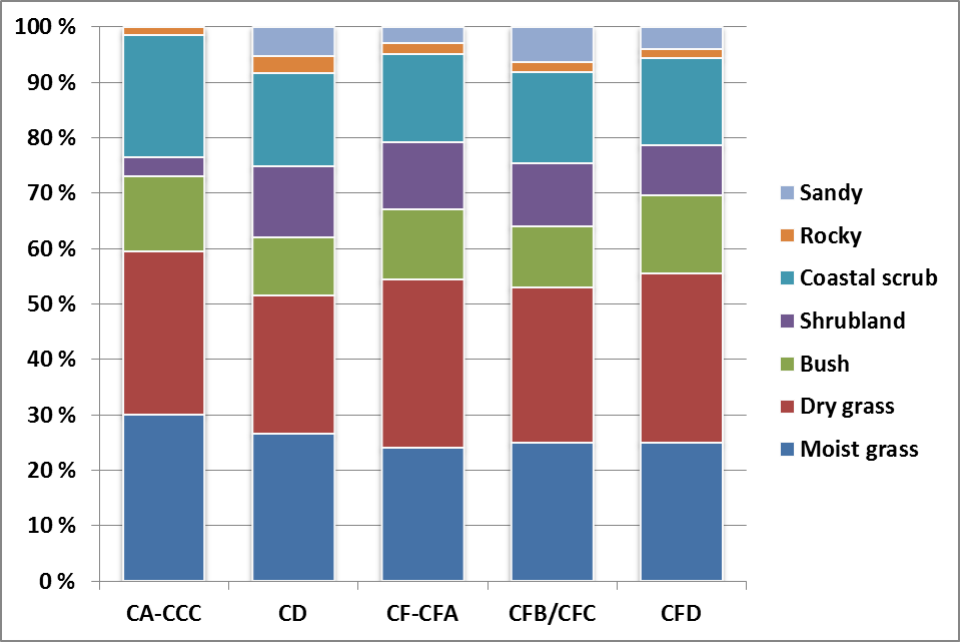
Reconstructed microhabitat at BBC during the SB based on the taxonomic habitat index. Results are given as stacked percentage distribution of the various vegetation and substrate types.

The weighting of the species present in the BBC micromammal assemblage are given in Table 7. Certain limits were set regarding the taxonomic representation used for calculation and only species identified to taxa were included, *O*. *irroratus* and *O*. *saundersiae* were both included when the category *O*. *irroratus*/*O*. *saundersiae* was present. The two species comprise the bulk of the *Otomys* sp. category but due to breakage of maxillae (which make up most of the *Otomys* sp. category) positive identification of taxon could not be done (for further discussion see Nel [115]). Chiroptera were omitted as their detailed vegetation preferences are difficult to establish. The index was weighted according to percentage abundance of species in order to obtain more nuanced information of the microhabitat. The results of the THI (Table 8 and Fig 10) are discussed below together with other palaeoclimatic evidence from the BBC region.

**Table 8:** Reconstructed microhabitat at BBC during the SB based on the taxonomic habitat index.

	Moist grass	Dry grass	Bush	Shrubland	Coastal scrub	Rocky	Sandy
**CA-CCC**	30.7	30	13.9	3.6	22.5	1.4	0
**CD**	26.5	24.7	10.5	12.6	16.8	3	5.2
**CF-CFA**	24	30.3	12.7	12	15.9	2	2.9
**CFB/CFC**	24.9	28.1	10.9	11.4	16.3	1.9	6.3
**CFD**	24.9	30.5	14.1	9	15.8	1.6	3.9

The results are given as percentage distribution of the various vegetation and substrate types

### Environmental interpretation

#### MIS 5a (CFD-CD)

Our palaeoenvironmental reconstruction indicates that the vegetation surrounding BBC in MIS 5a was a mixture of biotopes that provided habitats for a wide variety of micromammal species. In particular the micromammal sample from layer CD shows the highest diversity for BBC [115] and confirms the presence of a range of biotopes and ecotones in the area. BBC, which earlier in the MIS 5 had been occupied ephemerally and largely in agreement with site to shore distances, was seemingly more frequently used during MIS 5a [60, 115]. The coherence of human occupation frequencies and distance to the shore is also observed at Pinnacle Point (Fig 1) [20, 32]. Seen in correlation with the micromammal palaeoenvironmental data, it is likely that the increased intensive human occupation of BBC reflect an area with a wide availability of subsistence resources in MIS 5a but that these quite rapidly may have decreases in MIS 4 leading to the abandonment of the occupations at BBC. After 72 ka the next known occupation of the cave was after 2 ka [31].

Based on the THI, MIS 5a vegetation seems proportionally stabile with rather small detectable variations. The first occurrence of Still Bay lithic points (n = 21) is in MIS 5a [50]. Their appearance may be associated with a relative increase in bush vegetation particularly in layers CFD and CF-CFA (Fig 10). There is also an increase in shrubland during the same period (Fig 10), which would signify further dense vegetation cover. It is possible that these bifacial points were used in hunting large mammal fauna but also small ungulates such as the common duiker (*Sylvicapra grimmia*), which are found in bushy, closed vegetation [17, 60, 63]. Thompson and Henshilwood [60] have noted that smaller ungulates were exploited more commonly by the BBC hunters in the M2 Upper phase. Bifacial points may thus have served as weapons of choice for hunting in dense, bushy vegetation and the processing of small ungulates [50].

Approximately 80 km further east at Pinnacle Point (Fig 1), Bar-Matthews *et al*. [33] suggest that fynbos followed the coast line during the lowering of sea levels in the latter part of MIS 5. The exposed plains in front of the Pinnacle Point cave complex during low sea levels may have provided suitable habitats for fynbos expansion [33] and this scenario may have been the same at BBC as both have a shallow off-shore topography, with large plains being exposed as sea levels retract [32] (Fig 1). The distance from the cave to the shore at BBC was c. 2.33 km during the latter part of MIS 5a and this might have put increasing pressure on subsistence practices at BBC as shellfish resources moved further away from the site [18, 32]. Shrubland seemingly has a slight increase throughout MIS 5a at BBC (Fig 10). According to the THI shrubland vegetation comprised 9% of the immediate BBC area in CFD, while in CD the shrubland component is 12.6%. The relative increase of shrubland may be due to shrubs and bushes associated with the fynbos biotope expanding as sea levels retracted on the exposed plains in front of the cave.

Quick *et al*. [125] have analysed pollen, charcoal and sedimentological data from the Vankervelsvlei wetland in Wilderness (Fig 1) and suggest that there may have been greater dominance of fynbos in this area during the latter part of MIS 5. They associate the possibly greater fynbos dominance with increased rainfall seasonality and cooler temperatures. The general decline of Soricidae abundances at BBC throughout MIS 5a may be further indication of increased winter rainfall and lower summer temperatures along the southern coastal region at the onset of MIS 4.

#### MIS 5a/4 (CA-CCC)

Although the sample size in CA-CCC is smaller than in the MIS 5a layers, the rarefaction analysis (Fig 7) suggests that the variation in species richness between CA-CCC and CD is a result of changes in climate, and thus environmental conditions. The small micromammal sample size in layers CA, CB, CC and CCC may be due to intensive use of the cave by humans during this relatively short time span [19, 60, 115], albeit simultaneously with an apparent decline in available micromammal prey for the African barn owl. The relative abundance of bats supports this assumption and further indicates changes in the vegetation structure in the latter part of the SB sequence at BBC.

The diversity of species is greater in areas where the seasonal variation in precipitation is moderate [85]. The decline of micromammal species diversity in CA-CCC, comparable to the other SB layers, could be suggestive of increased seasonal precipitation at the MIS 5a/4 transition. Blome *et al*. [7] have noted that the strength and position of the westerlies likely had a significant impact on regional precipitation patterns in southern Africa. Previously established palaeo records from sites situated in the YRZ and WRZ suggest that MIS 4 was characterised by humid conditions [7, 37, 77, 93]. Quick *et al*. [125] inferred that the suggested cooler temperatures and perhaps increased rainfall seasonality (greater dominance of fynbos) in MIS 5 a/b persisted throughout much of the MIS 4, and coincide with a northward migration of the Subtropical Convergence and likely increases in winter rainfall and a decrease in summer rainfall [125]. Urrego *et al*. [129] have also noted, based on terrestrial and marine climatic tracers from a marine core sample, expansion of fynbos vegetation on the west coast of South Africa in MIS 4 and associated the expansion with increasing seasonal moisture during the austral winter months. The possible increase in winter rainfall at BBC might be linked to a regional onset of a more seasonally driven climate in MIS 4 compared to MIS 5.

**Fig 11:**
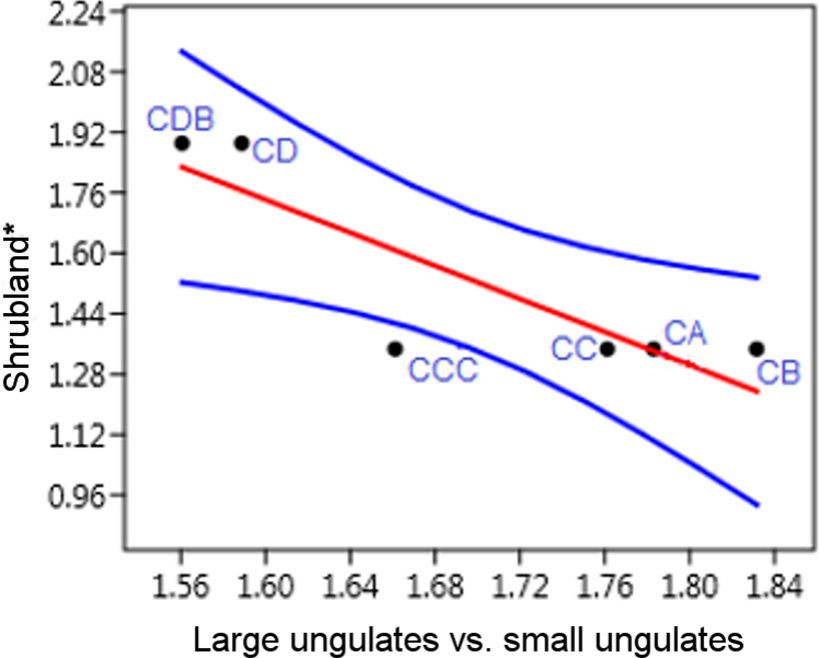
Linear relationship between proportional abundance of large vs. small ungulates and shrubland vegetation in the M1 phase at BBC. R = -0.86, R^2^ = 0.7, p<0.03, 95% confidence intervals. *Proportional abundance of vegetation component in CA-CCC and CD based on THI. The numbers of large and small ungulates are based on data presented in Discamps and Henshilwood [19].

The micromammal data indicates that shrubland vegetation declined during the occupation of the CA-CCC layers (Fig 10) and Fischer *et al*. [32] have estimated that the distance from the cave to the shore may have been as far as c. 15.56 km towards the end of the M1 phase. This does not necessarily stipulate a general decline in fynbos in the region, but rather highlights local variations in proportions of the various types of fynbos vegetation. The increase in moist grass in CA-CCC could be indicative of expanding reed vegetation endemic to the fynbos biome and the decline in shrubs associated with less nutrient rich soil. There were likely more Ericaceae and Proteaceae type fynbos vegetation on the exposed plain, while an increase in seasonal rainfall allowed for more moist grasses and generally greater grass and scrub cover near the cave. The large mammal data from the M1 phase show a reduction in small ungulates such as grysbok/steenbok (*Raphicerus* spp.) and bushbuck, species currently associated with fynbos in the Western Cape Province [19, 60, 91]. This coincides with the findings of Discamps and Henshilwood [19] who report an increase in the abundance of large ungulates during the M1 phase. There is a significant negative correlation (p<0.05) between the proportional abundance of large vs. small ungulates and shrubland vegetation (Fig 11). When shrubland vegetation declines, large ungulate proportional abundance becomes greater. The reduction of small ungulates in the upper layers of M1 may be due to a shift in biotopes in the vicinity of the cave, which would have led the human occupants to either expand their hunting strategies to other large mammals or to travel further onto the exposed coastal plain, away from the cave, to hunt for small browsers.

During the M1 phase, the MSA people at BBC experienced declines in the return rates from two of their major collectable animal resources; shellfish and tortoise [18]. At the MIS 5a/4 transition, the MSA humans probably experienced decreasing encounter rates and smaller sizes of the tortoises which were collected [18].The shellfish abundances declined (per m^3^ of deposit) as a likely result of the increasing distance to the coastline [17, 18, 32].

MSA hunting strategies during this period demonstrate their flexibility and successful response to changes in local conditions and resource availability, similar to modern hunting practices (e.g. Kelly [130], Thompson [63]). When shrubland declined, both grass habitats and scrub increased (Fig 10), and correspondingly human hunters began to hunt larger ungulates more often in the latter part of M1 [19, 60] (Fig 11). The micromammal environmental implications thus support a potential shift in prey availability where larger ungulates (size class 1 and 2) became more readily available [19].

Several researchers have associated the SB techno-tradition with climatic and by extension environmental alterations at the onset of MIS 4 [4, 8, 33, 37, 131]. It is likely that the micromammal composition in CA-CCC at BBC reflects rapid environmental change. The similarity indices (Fig 9) show that the uppermost layers in M1 differ from the other SB layers on a high rank level. Thus the habitats surrounding BBC changed to such an extent that some micromammal species disappeared.

BBC is generally more intensively used by humans during MIS 5a/4 comparatively to MIS 5 [19, 60, 115]. The intensive use of the cave coincides with expansion of grassland, more open vegetation and seasonal rainfall. Climatic conditions which negatively affected the micromammal community seem to have provided optimal conditions for large mammals and thus hunting of these animals by humans. The utilization of BBC signifies that the changes in climate at the onset of MIS 4 did not negatively affect the availability of some resources in the area but in the final phases it is clear that the site occupants were under increasing pressure from deteriorating climatic conditions.

## Conclusion

The Still Bay sequence at BBC marks a 5–6 ka (76–72 ka) period of high intensity human occupation of the site [60]. Here we discuss the palaeoenvironmental implications deduced from our micromammal study in the context of the human utilisation of the cave and as a comparison with other palaeoclimatic evidence from the region. We emphasise that although the period studied covers only 5–6 ka there are considerable changes in climate and palaeoenvironment during this time span. The effect of these changes on the humans that occupied the cave, mostly for brief periods, was over time quite considerable. The material culture from the lower SB levels at c. 76 ka shows distinct differences, than that found in the upper levels [29, 53]. There is a far greater intensity in the production of symbolic material culture that likely mediated the behaviour of the SB people at BBC [29]. One reason for the intensity of production of engraved ochres, manufacture of finely made, aesthetically pleasing, bifacial points crafted through pressure flaking on heat-treated silcrete, plus the rapid increase in the number of marine shell beads in the upper layers, is that, as the colder conditions of MIS 4 approached, these people were under pressure to survive in increasingly adverse conditions. One way of signalling this pressure among MSA people was probably increasing the production of symbolically mediated artefacts and technological complexity, perhaps for exchange with other groups and is also a signal that groups within regions could have required greater co-operation to survive. After 72 ka the Still Bay techno-tradition abruptly disappears from southern Africa and is replaced by the Howiesons Poort only about 5 ka later. Just after 72 ka the entrance to BBC is sealed with aeolian sand and the cave is no longer accessible for human habitation. This rapid movement of sand also provides us with clear evidence of the fast changing environmental conditions after about 72 ka that coincides with a lowering of sea levels. It is in this setting that we have placed our environmental study based on the micromammals from the SB levels. The results confirm our other findings of environmental and cultural change from 76–72 ka [18, 29]. This 5–6 ka period of the MSA is thus highly significant in the evolution of *H*. *sapiens* in this region and our study here adds new data to our current knowledge of these early humans and the demise of the Still Bay techno-tradition [19, 52, 53].

## Supporting Information

S1 Table Standardisation of individual rarefaction curves. (DOCX)
