# Supplementary material for: The Small Mammal Sequence from the c. 76 – 72 ka Still Bay Levels at Blombos Cave, South Africa – Taphonomic and Palaeoecological Implications for Human Behaviour
Source: PLoS One. 2016 Aug 10;11(8):e0159817. doi: 10.1371/journal.pone.0159817 (PMC4980004; doi:10.1371/journal.pone.0159817)
Supplement: S1 Table — (DOCX) [file pone.0159817.s001.docx]

**Supporting information**

**S1 Table: Standardisation of individual rarefaction curves.**

| **Phase** | **Units** | **Species richness**** | **Standard deviation** | **Mean** | ***t* test** | **p value** | **Permutation *t* test** |
| --- | --- | --- | --- | --- | --- | --- | --- |
| **M1** | CA-CCC* | 9.00 | 0.00 | 6.37 |  |  |  |
|  | CD | 12.56 | 1.45 | 8.07 | -2.16 | **0.035** | 0.035 |
| **M2 Upper** | CF-CFA | 10.92 | 1.09 | 7.10 | -1.03 | 0.308 | 0.308 |
|  | CFB/CFC | 11.79 | 1.27 | 9.88 | -1.64 | 0.107 | 0.107 |
|  | CFD | 10.71 | 1.12 | 7.10 | -1.04 | 0.302 | 0.302 |

Results of *t*-test and permutation test (n=9999) for the M1 and M2 Upper fossil micromammal assemblage at Blombos Cave.

*Standardised sample, MNI = 27.

**When all samples are standardised to MNI = 27.
